# Interactions of the Apolipoprotein A5 Gene Polymorphisms and Alcohol Consumption on Serum Lipid Levels

**DOI:** 10.1371/journal.pone.0017954

**Published:** 2011-03-14

**Authors:** Rui-Xing Yin, Yi-Yang Li, Wan-Ying Liu, Lin Zhang, Jin-Zhen Wu

**Affiliations:** 1 Department of Cardiology, Institute of Cardiovascular Diseases, The First Affiliated Hospital, Guangxi Medical University, Nanning, Guangxi, People's Republic of China; 2 Department of Cardiology, Guangxi National Hospital, Nanning, Guangxi, People's Republic of China; University of Michigan, United States of America

## Abstract

**Background:**

Little is known about the interactions of apolipoprotein (Apo) A5 gene polymorphisms and alcohol consumption on serum lipid profiles. The present study was undertaken to detect the interactions of ApoA5–1131T>C, c.553G>T and c.457G>A polymorphisms and alcohol consumption on serum lipid levels.

**Methodology/Principal Findings:**

A total of 516 nondrinkers and 514 drinkers were randomly selected from our previous stratified randomized cluster samples. Genotyping was performed by polymerase chain reaction and restriction fragment length polymorphism. The levels of serum total cholesterol (TC), triglyceride (TG), high-density lipoprotein cholesterol (HDL-C), ApoA1 and ApoB were higher in drinkers than in nondrinkers (*P*<0.05–0.001). The genotypic and allelic frequencies of three loci were not different between the two groups. The interactions between –1131T>C genotypes and alcohol consumption on ApoB levels (*P*<0.05) and the ApoA1/ApoB ratio (*P*<0.01), between c.553G>T genotypes and alcohol consumption on low-density lipoprotein cholesterol (LDL-C) levels (*P*<0.05) and the ApoA1/ApoB ratio (*P*<0.05), and between c.457G>A genotypes and alcohol consumption on TG levels (*P*<0.001) were detected by factorial regression analysis after controlling for potential confounders. Four haplotypes (T-G-G, C-G-G, T-A-G and C-G-T) had frequencies ranging from 0.06 to 0.87. Three haplotypes (C-G-G, T-A-G, and C-G-T) were significantly associated with serum lipid parameters. The –1131T>C genotypes were correlated with TG, and c.553G>T and c.457G>A genotypes were associated with HDL-C levels in nondrinkers (*P*<0.05 for all). For drinkers, the –1131T>C genotypes were correlated with TC, TG, LDL-C, ApoB levels and the ApoA1/ApoB ratio (*P*<0.01 for all); c.553G>T genotypes were correlated with TC, TG, HDL-C and LDL-C levels (*P*<0.05–0.01); and c.457G>A genotypes were associated with TG, LDL-C, ApoA1 and ApoB levels (*P*<0.05–0.01).

**Conclusions:**

The differences in some serum lipid parameters between the drinkers and nondrinkers might partly result from different interactions of the ApoA5 gene polymorphisms and alcohol consumption.

## Introduction

Dyslipidemia has become a major health problem in many countries because of its high prevalence and a causal relationship with serious medical condition such as coronary artery disease (CAD), hypertension and stroke [1]. It is well known that dyslipidemia is a complex trait caused by multiple environmental and genetic factors and their interactions [2]–[6]. Numerous studies have evaluated the influence of alcohol consumption on CAD and plasma lipid concentrations. Low to middle amounts of alcohol when taken on a regular basis have been shown to protect against CAD and death [7], [8], whereas heavy drinking constitutes a severe risk condition. A moderate intake of alcohol is associated with protection against CAD, probably due in part to a dose-dependent increase in high-density lipoprotein cholesterol (HDL-C) [9]–[11]. A decrease in low-density lipoprotein cholesterol (LDL-C) with increased alcohol intake has also been reported in some studies, but this effect is less consistent and probably depends on the combination of one or more unmeasured factors [12]. However, alcohol in doses >30 g/day in both sexes can augment serum triglyceride (TG) levels. The alcohol intake of 60 g/day increases the TG levels by about 0.19 mg/dl per 1 gram of alcohol consumed [13].

Apolipoprotein (Apo) A5 is a secreted protein present in human serum and is associated with specific lipoprotein particles. It was detectable in very low-density lipoprotein (VLDL), HDL, and chylomicrons. Serum ApoA5 is very low compared with other apolipoproteins. Human serum ApoA5 concentrations range from 24 to 406 µg/l, with a mean value in normolipidemic persons of 157 µg/l [14], or 179.2±74.8 ng/ml in the healthy subjects [15]. ApoA5 is a key regulator of serum TG concentrations. The gene of ApoA5 was originally identified by experiments looking for new open reading frames in the ApoA1-ApoC3-ApoA4 gene cluster, which is located on human chromosome 11q23 [16], [17]. The human ApoA5 gene consists of four exons and three introns and codes for a 369 amino acid protein, ApoA5. It has been reported that the human ApoA5 transgenic mouse has significantly decreased TG and the ApoA5 gene knockout mouse significantly increased plasma TG concentrations as compared with wild-type mice [17], [18]. The adenovirus-mediated overexpression of ApoA5 was associated with markedly decreased (–70%) serum TG levels caused primarily by the reduced TG content of the VLDL fraction [19]. These data suggested that ApoA5 expression may be highly and inversely correlated with TG concentrations. At least 150 single nucleotide polymorphisms (SNPs) have been identified in the ApoA5 gene (http://www.ncbi.nlm.nih.gov/SNP/). However, for most of them there are no robust data about their functional relevance. The minor alleles of several common SNPs in the human ApoA5 gene locus have been reported to be significantly associated with increased plasma TG levels [20]–[44] and the risk of CAD [45]–[51] in some studies but not in others [52], [53]. We hypothesize that these conflicting results in diverse populations might partly result from different gene-environment interactions. However, little is known about the interactions of ApoA5 gene polymorphisms and alcohol consumption on serum lipid concentrations. Therefore, the aim of the present study was to detect the interactions of ApoA5 –1131T>C (rs662799), c.553G>T (rs2075291) and c.457G>A (rs3135507) polymorphisms and alcohol consumption on serum lipid levels.

## Methods

### Study subjects

A total of 1030 unrelated subjects who reside in 16 villages in Napo County, Guangxi Zhuang Autonomous Region, People's Republic of China were randomly selected from our previous stratified randomized cluster samples [2]. The age of the subjects ranged from 15 to 89 years, with an average age of 43.30±17.69 years. There were 516 nondrinkers and 514 drinkers. All of the subjects were rural agricultural workers. The subjects had no evidence of diseases related to atherosclerosis, CAD and diabetes. None of them had been treated with β-adrenergic blocking agents and lipid-lowering drugs such as statins or fibrates. The present study was approved by the Ethics Committee of the First Affiliated Hospital, Guangxi Medical University. Verbal informed consents and their thumbprints (fingerprints, to express consent) were obtained from all subjects after they received a full explanation of the study. Written informed consents were not obtained because the educational level of the subjects was very low. The procedure was also approved the Ethics Committee. An incentive of about ten dollars was provided to each participant in the study.

### Epidemiological survey

The survey was carried out using internationally standardized methods, following a common protocol [54]. Information on demographics, socioeconomic status, and life style was collected with standardized questionnaires. Smoking status was categorized into groups of cigarettes per day: <20 and ≥20. Alcohol consumption was categorized into groups of grams of alcohol per day: ≤25 and >25. The physical examination was conducted by a trained/licensed cardiovascular physician. Blood pressure, body height, and body weight were measured, and body mass index (BMI) was calculated as weight (kg) divided by height (m) squared. Sitting blood pressure was measured three times with use of a mercury sphygmomanometer after the subjects had a 5 min rest, and the average of the three measurements was used for statistical analysis. Systolic blood pressure was determined by the first Korotkoff sound, and diastolic blood pressure by the fifth Korotkoff sound.

### Biochemical analysis

Venous blood samples (8 ml) were drawn from a forearm vein of every subject after venous occlusion for a few seconds in a sitting position, after an overnight fast of 12 h and abstention from alcohol use for at least 12 h. A part of the sample (3 ml) was collected into glass tubes and allowed to clot at ambient temperature, and used to determine serum lipid levels, and another part of the sample (5 ml) was transferred into tubes with anticoagulate solution (4.80 g/l citric acid, 14.70 g/l glucose, and 13.20 g/l tri-sodium citrate) and used to extract deoxyribonucleic acid (DNA). Immediately following clotting serum was separated by centrifugation for 15 min at 3000 rpm. The levels of serum total cholesterol (TC), TG, HDL-C, and LDL-C in samples were determined by enzymatic methods with commercially available kits, Tcho-1, TG-LH (RANDOX Laboratories Ltd., Ardmore, Diamond Road, Crumlin Co. Antrim, United Kingdom, BT29 4QY), Cholestest N HDL, and Cholestest LDL (Daiichi Pure Chemicals Co., Ltd., Tokyo, Japan); respectively. Serum ApoA1 and ApoB levels were assessed by the immunoturbidimetric immunoassay using a commercial kit (RANDOX Laboratories Ltd.). All determinations were performed with an autoanalyzer (Type 7170A; Hitachi Ltd., Tokyo, Japan) in the Clinical Science Experiment Center of the First Affiliated Hospital, Guangxi Medical University.

### DNA amplification and genotyping

Total genomic DNA was isolated from peripheral blood leukocytes using the phenol-chloroform method [5], [6]. The extracted DNA was stored at 4°C until analysis. Genotyping of the ApoA5 –1131T>C, c.553G>T and c.457G>A was performed by polymerase chain reaction and restriction fragment length polymorphism (PCR-RFLP) according to the previous reports [16], [31], [50]. For the genotyping of ApoA5 –1131T>C, the sequence of the forward and backward primers used was 5′-GATTGATTCAAGATGCATTTAGGAC-3′ and 5′-CCCCAGGAACTGGAGCGAAATT-3′ (Sangon, Shanghai, People's Republic of China). Each reaction system of a total volume of 25 µl, comprised 0.2 µg of genomic DNA; 0.8 µl of each primer (10 pmol/µl); 4.0 µl of 10×buffer solution; 1.5 µl of MgCl_2_ (25 mmol/l); 2.0 µl of dNTP (2.5 mmol/l); and 1.5 U of *Taq* polymerase (Takara). For the amplification, initial denaturation at 95°C for 5 min was followed by 30 cycles of denaturation at 95°C for 30 s, annealing at 61°C for 30 s, and extension at 72°C for 45 s, with final extension at 72°C for 7 min. Eight microliters of the PCR product were digested with 3 U of *Tru1*I (*Mse*I) at 65°C for 8 h. The digestive products were separated by electrophoresis on 2% sepharose gel for 60 min. Both c.553G>T and c.457G>A are naturally occurring restriction enzyme sites in the exon 4 of ApoA5 gene. To analyze these two polymorphic markers, exon 4 was amplified using primers 5′-TCGGCGTATGGGTGGAAGAG-3′ and 5′-GGCAGCAACTGAAGCCCTACAC-3′ (Sangon, Shanghai, People's Republic of China). Each reaction system of a total volume of 25 µl, comprised 0.2 µg of genomic DNA; 0.8 µl of each primer (10 pmol/µl); 4.0 µl of 10×buffer solution; 1.5 µl of MgCl_2_ (25 mmol/l); 2.0 µl of dNTP (2.5 mmol/l); and 1.5 U of *Taq* polymerase (Takara). For the amplification, initial denaturation at 95°C for 5 min was followed by 30 cycles of denaturation at 95°C for 30 s, annealing at 61°C for 30 s, and extension at 72°C for 45 s, with final extension at 72°C for 7 min. Each restriction enzyme reaction was performed with 8 µl of amplified DNA; 1 µl of 10×buffer solution; and 4 U *Msp*I (c.553G>T) or 4 U *Nsb*I (*Fsp*I, c.457G>A) restriction ezyme in a total volume of 20 µl digested at 37°C overnight. The length of each digested DNA fragment was determined by comparing migration of a sample with that of standard DNA marker. Stained with ethidium bromide, the gel was visualized under ultraviolet light and photographed. Genotypes were scored by an experienced reader blinded to epidemiological and lipid results.

### DNA sequencing

Sixteen samples (each genotype in two) detected by the PCR-RFLP were also confirmed by direct sequencing. The PCR product was purified by low melting point gel electrophoresis and phenol extraction, and then the DNA sequences were analyzed by using an ABI Prism 3100 (Applied Biosystems) in Shanghai Sangon Biological Engineering Technology & Services Co., Ltd., People's Republic of China.

### Diagnostic criteria

The normal values of serum TC, TG, HDL-C, LDL-C, ApoA1, ApoB levels and the ratio of ApoA1 to ApoB in our Clinical Science Experiment Center were 3.10–5.17, 0.56–1.70, 0.91–1.81, 1.70–3.20 mmol/L, 1.00–1.76, 0.63–1.14 g/L, and 1.00–2.50; respectively [2], [3]. Hypertension was diagnosed according to the criteria of 1999 The World Health Organization-International Society of Hypertension Guidelines for the management of hypertension [55], [56]. The diagnostic criteria of overweight and obesity were according to the Cooperative Meta-analysis Group of China Obesity Task Force. Normal weight, overweight and obesity were defined as a BMI < 24, 24–28, and >28 kg/m^2^; respectively [57].

### Statistical analysis

The data were recorded on a pre-designed form and managed with Excel software. The quantitative variables were presented as mean ± standard deviation (serum TG levels were presented as medians and interquartile ranges). The difference in general characteristics between the two groups was tested by the Student's unpaired *t* test. The frequency of the ApoA5 alleles was determined by gene counting. A chi-square analysis was used to evaluate the allelic and genotypic frequencies that were calculated from the observed genotypic counts, and standard goodness-of-fit test was used to test the Hardy-Weinberg equilibrium. The interactions between the ApoA5 genotypes and alcohol consumption on serum lipid parameters were assessed by using a factorial regression analysis after controlling for potential confounders including sex, age, BMI, hypertension, and cigarette smoking. Pair-wise linkage disequilibria (LD) among the three SNPs were estimated as correlation coefficients (*r* ^2^) using the HelixTree program (GOLDEN Helix, Bozeman, MN, USA). For haplotype analysis, we estimated haplotype frequencies using the expectation-maximization algorithm, and determine the association between haplotypes and lipid phenotypes using trend regression analysis with the option of composite haplotype estimation implemented in HelixTree. *P* values were further adjusted for multiple tests by a permutation test. The permutation test was conducted by changing the orders of dependant variable randomly against the genotypes. Then haplotype trend regression was conducted based on the same model and a *P* value was recorded. In order to evaluate the association of serum lipid parameters with several environmental factors, alleles (–1131C allele noncarriers  =  1, carriers  =  2) and genotypes (–1131T>C: TT  =  1, TC  =  2, CC  =  3; c.553G>T: GG  =  1, GT  =  2; c.457G>A: GG  =  1, GA/AA  =  2; respectively), multiple linear regression analysis with forward stepwise modeling was also performed in the combined population, nondrinkers, and drinkers; respectively. The statistical analyses were performed with the statistical software package SPSS 13.0 (SPSS Inc., Chicago, Illinois). A *P* value of less than 0.05 was considered statistically significant.

## Results

### General characteristics between nondrinkers and drinkers

Table 1 gives the general characteristics between the nondrinkers and drinkers. The ratio of male to female, the mean age, the levels of systolic blood pressure, diastolic blood pressure and pulse pressure, and the percentages of subjects who smoked cigarettes were higher in drinkers than in nondrinkers (*P*<0.05–0.001). There was no significant difference in the BMI between the two groups (*P*>0.05).

**Table 1:** Comparison of the general characteristics and serum lipid levels between the nondrinkers and drinkers.

Parameter	Nondrinker(n = 516)	Drinker(n = 514)	*t* (*x* ^2^)	*P*
Male/female	186/330	306/208	56.930	0.000
Age (years)	41.26±19.53	45.35±15.38	–3.733	0.000
Body mass index (kg/m^2^)	21.66±2.71	21.95±2.39	–1.821	0.069
Systolic blood pressure (mmHg)	120.03±16.15	125.60±15.63	–5.624	0.000
Diastolic blood pressure (mmHg)	74.13±9.82	77.79±9.78	–5.993	0.000
Pulse pressure (mmHg)	45.95±11.89	47.83±12.01	–2.525	0.012
Cigarette smoking [n (%)]				
Nonsmoker	414(80.2)	292(56.8)		
<20 cigarettes/day	62(12.0)	106(20.6)		
≥20 cigarettes/day	40(7.8)	116(22.6)	69.628	0.000
Alcohol consumption [n (%)]				
Nondrinker	516(100.0)	–		
<25 g/day	–	396(77.0)		
≥25 g/day	–	118(23.0)		
Total cholesterol (mmol/L)	4.52±0.99	4.65±0.95	–2.150	0.032
Triglyceride (mmol/L)	0.97(0.57)	1.09(0.61)	–2.488	0.013
HDL-C (mmol/L)	1.98±0.45	2.14±0.49	–5.458	0.000
LDL-C (mmol/L)	2.40±0.70	2.41±0.70	–0.229	0.819
Apolipoprotein (Apo) A1 (g/L)	1.40±0.16	1.48±0.13	–8.805	0.000
ApoB (g/L)	0.89±0.22	0.92±0.20	–2.290	0.022
ApoA1/ApoB	1.68±0.57	1.69±0.46	–0.310	0.757

HDL-C, high-density lipoprotein cholesterol; LDL-C, low-density lipoprotein cholesterol. The value of TG was presented as median (interquartile range). The difference between the two groups was determined by the Wilcoxon-Mann-Whitney test.

### Serum lipid levels between nondrinkers and drinkers

The levels of TC, TG, HDL-C, ApoA1 and ApoB were higher in drinkers than in nondrinkers (*P*<0.05–0.001). There was no significant difference in the levels of LDL-C and the ratio of ApoA1 to ApoB between the two groups (*P*>0.05 for each).

### Results of electrophoresis and genotyping

**Figure 1:**
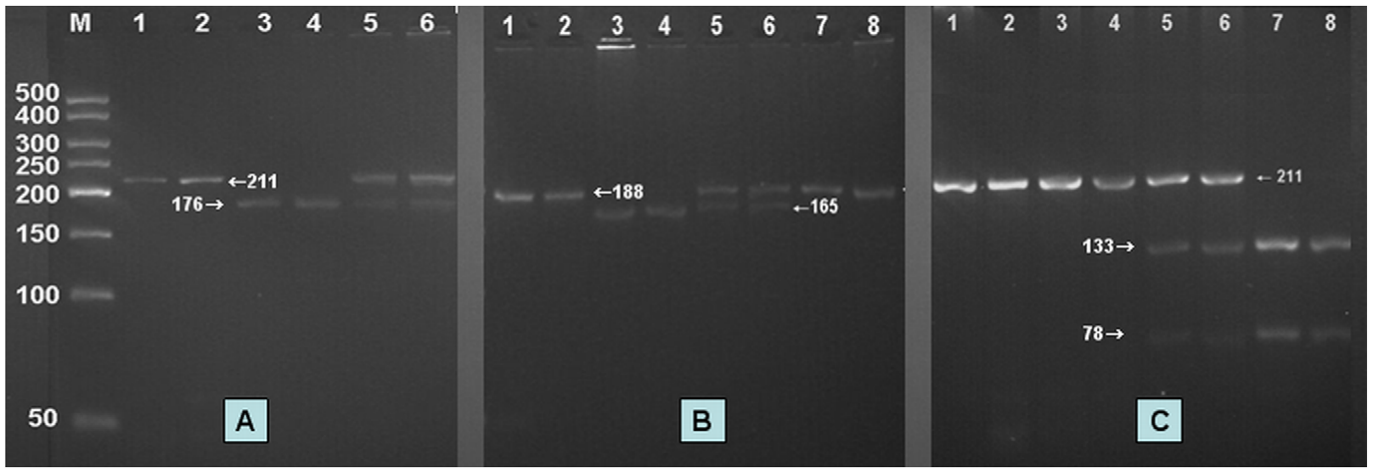
Genotyping of the ApoA5 gene polymorphisms. (A) ApoA5 c.553G>T. Lane M, 50 bp marker ladder; lanes 1 and 2, the PCR products of the samples (211 bp); lanes 3 and 4, GG genotype (176 bp and 35 bp); lanes 5 and 6, GT genotype (211 bp, 176 bp and 35 bp). TT genotype was not be detected in both groups. The 35 bp fragment was invisible in the gel owing to its fast migration speed. (B) ApoA5 –1131T>C. Lanes 1 and 2, the PCR products of the samples (188 bp); lanes 3 and 4, TT genotype (165 bp and 23 bp); lanes 5 and 6, TC genotype (188 bp, 165 bp and 23 bp); and Lanes 7 and 8, CC genotype (188 bp). The 23 bp fragment was invisible in the gel owing to its fast migration speed. (C) ApoA5 c.457G>A. Lanes 1 and 2, the PCR products of the samples (211 bp); lanes 3 and 4, GG genotype (211 bp); lanes 5 and 6, GA genotype (211 bp, 133 bp and 78 bp); and lanes 7 and 8, AA genotype (133 bp and 78 bp).

After the genomic DNA of the samples was amplified by PCR and imaged by 2% agarose gel electrophoresis for the ApoA5 c.553G>T, the PCR product of 211 bp nucleotide sequences could be seen in the samples. The GG and GT genotypes were shown in Figure 1A. The TT genotype was not detected in our study population. The PCR product of the ApoA5 –1131T>C was 188 bp nucleotide sequences. The TT, TC and CC genotypes were shown in Figure 1B. The PCR product of the ApoA5 c.457G>A was 211 bp nucleotide sequences. The GG, GA and AA genotypes were shown in Figure 1C.

### Results of sequencing

**Figure 2:**
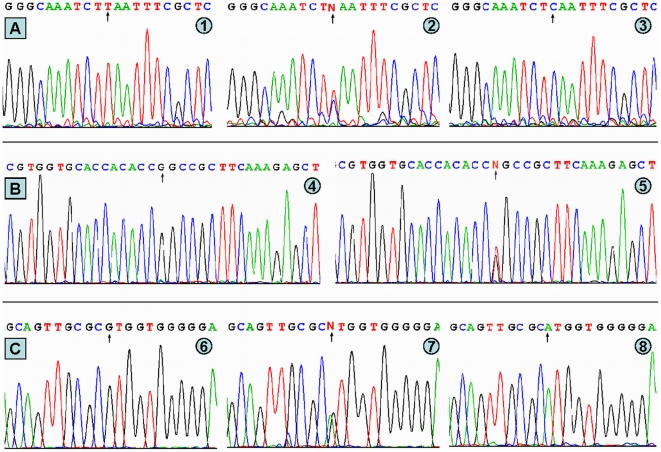
A part of the nucleotide sequences of the ApoA5 gene polymorphisms. (A) ApoA5 –1131T>C: (1) TT genotype, (2) TC genotype, (3) CC genotype; (B) ApoA5 c.553G>T: (4) GG genotype, (5) GT genotype; (C) ApoA5 c.457G>A: (6) GG genotype, (7) GA genotype, (8) AA genotype.

The results were shown as TT, TC and CC genotypes of the –1131T>C, GG and GT genotypes of the c.553G>T, and GG, GA and AA genotypes of the c.457G>A by PCR-RFLP, the genotypes were also confirmed by sequencing (Figure 2); respectively.

### Genotypic and allelic frequencies

The genotypic and allelic frequencies of the three SNPs are shown in Table 2. The genotypic frequencies of each of the three loci were all in Hardy-Weinberg equilibrium (–1131T>C: *x* ^2^ = 1.524, *P* = 0.217; c.553G>T: *x* ^2^ = 1.200, *P* = 0.273; c.457G>A: *x* ^2^ = 2.470, *P* = 0.116; respectively). For three SNPs, –1131T>C was in LD with c.553G>T (LD coefficient  =  0.245, *P*<0.001) and c.457G>A (LD = 0.165, *P*<0.001). But c.553G>T variant was not in LD with c.457G>A (LD = 0.048, *P* = 0.126). There was no difference in the genotypic and allelic frequencies of the three SNPs between the nondrinkers and drinkers (*P*>0.05 for all).

**Table 2:** The genotypic and allelic frequencies of ApoA5 gene polymorphisms between the nondrinkers and drinkers [n (%)].

SNP	Group	n	Genotype [n (%)]			Allele [n (%)]	
			AA AB BB			A B	
–1131T>C	Nondrinker	516	268(51.9)	208(40.3)	40(7.8)	744(72.1)	288(27.9)
(rs662799)	Drinker	514	266(51.8)	196(38.1)	52(10.1)	728(70.8)	300(29.2)
	*x* ^2^	–	1.925			0.411	
	*P*	–	0.382			0.521	
	Drinker						
	<25 g/day	396	206(52.0)	152(38.4)	38(9.6)	564(71.2)	228(28.8)
	≥25 g/day	118	60(50.8)	44(37.3)	14(11.9)	164(69.5)	72(30.5)
	*x* ^2^	–	0.515			0.261	
	*P*	–	0.773			0.609	
c.553G>T	Nondrinker	516	482(93.4)	34(6.6)	–	998(96.7)	34(3.3)
(rs2075291)	Drinker	514	480(93.4)	34(6.6)	–	994(96.7)	34(3.3)
	*x* ^2^	–	0.000			0.000	
	*P*	–	0.988			0.988	
	Drinker						
	<25 g/day	396	370(93.4)	26(6.6)	–	766(96.7)	26(3.3)
	≥25 g/day	118	110(93.2)	8(6.8)	–	228(96.6)	8(3.4)
	*x* ^2^	–	0.008			0.007	
	*P*	–	0.934			0.936	
c.457G>A	Nondrinker	516	454(88.0)	60(11.6)	2(0.4)	968(93.8)	64(6.2)
(rs3135507)	Drinker	514	454(88.3)	52(10.1)	8(1.6)	960(93.4)	68(6.6)
	*x* ^2^	–	4.168			0.147	
	*P*	–	0.125			0.701	
	Drinker						
	<25 g/day	396	354(89.4)	36(9.1)	6(1.5)	744(93.9)	48(6.1)
	≥25 g/day	118	100(84.7)	16(13.6)	2(1.7)	216(91.5)	20(8.5)
	*x* ^2^	–	2.036			1.715	
	*P*	–	0.361			0.190	

Allele A, –1131T, c.553G or c.457G; Allele B, –1131C, c.553T or c.457A; Genotype AA, –1131TT, c.553GG or c.457GG; Genotype AB, –1131TC, c.553GT or c.457GA; Genotype BB, –1131CC, c.553TT or c.457AA.

### Interactions between genotypes and alcohol on lipid parameters

Table 3 shows the interactions between the ApoA5 genotypes and alcohol consumption on serum lipid parameters. Interactions between –1131T>C genotypes and alcohol consumption on serum ApoB levels (*P*<0.05) and the ratio of ApoA1 to ApoB (*P*<0.01) were detected, the subjects with CC genotype had higher serum ApoB levels and lower the ratio of ApoAI to ApoB in drinkers than in nondrinkers. Interactions between c.553G>T genotypes and alcohol consumption on serum LDL-C levels (*P*<0.05) and the ratio of ApoA1 to ApoB (*P*<0.05) were found, the subjects with GT genotype had lower serum LDL-C levels and higher the ratio of ApoAI to ApoB in drinkers than in nondrinkers. Interaction between c.457G>A genotypes and alcohol consumption on serum TG levels (*P*<0.001) was determined, the subjects with GA/AA genotype had lower serum TG levels in drinkers than in nondrinkers.

**Table 3:** The interactions between the ApoA5 genotypes and alcohol consumption on serum lipid parameters detected by factorial regression analysis.

SNP	n	TC	TG	HDL-C	LDL-C	ApoA1	ApoB	ApoA1/ApoB
ApoA5 –1131T>C								
Nondrinker TT	268	4.48±0.92	0.91(0.56)	1.98±0.41	2.37±0.71	1.40±0.13	0.88±0.21	1.71±0.61
TC	208	4.55±1.13	1.00(0.62)	1.97±0.50	2.43±0.72	1.40±0.19	0.91±0.22	1.62±0.42
CC	40	4.64±0.60	0.99(0.57)	2.00±0.44	2.45±0.60	1.41±0.12	0.87±0.25	1.83±0.87
Drinker TT	266	4.68±0.92	0.89(0.53)	2.15±0.47	2.35±0.72	1.47±0.14	0.89±0.20	1.74±0.50
TC	196	4.83±0.82	1.12(0.73)	2.13±0.51	2.42±0.65	1.48±0.13	0.94±0.20	1.66±0.42
CC	52	5.17±0.93	1.38(0.94)	2.15±0.55	2.66±0.69	1.50±0.10	1.03±0.20^b^	1.52±0.30^b^
* F*	–	1.143	0.518	0.075	1.006	0.259	4.273	5.059
* P*	–	0.319	0.596	0.928	0.366	0.772	0.014	0.007
ApoA5 c.553G>T								
Nondrinker GG	482	4.51±1.01	0.93(0.58)	1.99±0.46	2.39±0.71	1.40±0.16	0.89±0.22	1.69±0.58
GT	34	4.60±0.78	1.05(0.62)	1.83±0.32	2.55±0.67	1.40±0.11	0.98±0.20	1.48±0.32
Drinker GG	480	4.80±0.89	1.00(0.60)	2.16±0.50	2.42±0.70	1.48±0.13	0.92±0.20	1.69±0.44
GT	34	4.63±0.90	1.14(1.29)	1.93±0.37	2.23±0.63^a^	1.45±0.15	0.92±0.27	1.73±0.59^a^
* F*	–	1.155	1.214	0.476	4.084	0.770	3.600	3.990
* P*	–	0.283	0.271	0.491	0.044	0.380	0.058	0.046
ApoA5 c.457G>A								
Nondrinker GG	454	4.55±0.97	0.97(0.58)	1.99±0.45	2.41±0.69	1.40±0.15	0.90±0.22	1.67±0.57
GA/AA	62	4.31±1.17	0.99(0.63)	1.88±0.46	2.30±0.79	1.37±0.22	0.85±0.24	1.74±0.58
Drinker GG	454	4.81±0.88	1.01(0.60)	2.17±0.50	2.44±0.71	1.49±0.13	0.93±0.21	1.69±0.47
GA/AA	60	4.63±0.97	0.95(0.49)^b^	1.94±0.40	2.22±0.57	1.41±0.14	0.87±0.17	1.70±0.45
* F*	–	0.102	15.663	1.648	0.554	2.329	0.167	0.288
* P*	–	0.750	0.000	0.200	0.457	0.127	0.683	0.591

SNP, single nucleotide polymorphism; TC, total cholesterol; TG, triglyceride; HDL-C, high-density lipoprotein cholesterol; LDL-C, low-density lipoprotein cholesterol; ApoA1, apolipoprotein A1; ApoB, apolipoprotein B; ApoA1/ApoB, the ratio of apolipoprotein A1 to apolipoprotein B.

The values of *F* and *P* are the interactions between the ApoA5 genotypes and alcohol consumption on serum lipid parameters; sex, age, BMI, hypertension, and cigarette smoking have been controlled for the statistical analyses.

a *P*<0.05 and ^b^ *P*<0.01 in comparison with the same genotype of the nondrinker.

### Haplotype analysis

We observed four haplotypes: T-G-G, C-G-G, T-A-G, and C-G-T with frequencies ranging from 0.06 to 0.87, representing 100% of all haplotypes in the both groups (Table 4). The results showed that ApoA5 haplotypes were significantly (*P*<0.05) associated at the global level with serum TC, TG, HDL-C, LDL-C, ApoA1, and ApoB levels, even after correction for multiple testing with permutation test. In particular, carriers of haplotype C-G-G had significantly higher serum TC, TG, LDL-C and ApoB levels and lower the ratio of ApoA1 to ApoB than noncarriers; carriers of haplotype T-A-G had significantly lower serum TC, HDL-C, LDL-C, ApoA1 and ApoB levels than noncarriers; and carriers of haplotype C-G-T had higher TG and lower HDL-C levels than noncarriers (Table 4).

**Table 4:** Association between ApoA5 haplotypes and serum lipid traits in the combined nondrinkers and drinkers.

Lipid	Haplotype	T-G-G	C-G-G	T-A-G	C-G-T	Haplotype global association	
–	Frequency	0.65	0.26	0.06	0.03	–	–
–	Frequency (carrier vsnoncarrier)	898 vs 132	454 vs 576	122 vs 908	62 vs 968	*P*	*P* afterpermutation correction
TC	Carrier	4.65±0.04	4.75±0.05	4.50±0.09	4.59±0.12		
(mmol/L)	Noncarrier	4.71±0.08	4.58±0.05	4.71±0.04	4.69±0.04		
	*P*	0.482	0.003	0.017	0.397	<0.001	0.001
TG	Carrier	1.21 (0.06)	1.32 (0.07)	1.58 (0.12)	1.41 (0.16)		
(mmol/L)	Noncarrier	1.36 (0.11)	1.16 (0.07)	1.19 (0.06)	1.21 (0.06)		
	*P*	0.058	<0.001	0.479	0.006	< 0.001	0.001
HDL-C	Carrier	2.12±0.02	2.11±0.03	1.99±0.05	1.92±0.06		
(mmol/L)	Noncarrier	2.04±0.04	2.10±0.03	2.13±0.02	2.12±0.02		
	*P*	0.065	0.725	0.003	<0.001	< 0.001	0.001
LDL-C	Carrier	2.37±0.03	2.44±0.04	2.23±0.07	2.33±0.09		
(mmol/L)	Noncarrier	2.44±0.06	2.32±0.04	2.42±0.03	2.40±0.03		
	*P*	0.242	0.004	0.005	0.440	0.002	0.002
ApoA1	Carrier	1.46±0.01	1.46±0.01	1.42±0.01	1.44±0.02		
(g/L)	Noncarrier	1.44±0.01	1.46±0.01	1.47±0.01	1.47±0.01		
	*P*	0.133	0.769	0.001	0.160	0.001	0.002
ApoB	Carrier	0.90±0.01	0.92±0.01	0.86±0.02	0.94±0.03		
(g/L)	Noncarrier	0.92±0.02	0.88±0.01	0.92±0.01	0.91±0.01		
	*P*	0.401	0.001	0.003	0.259	< 0.001	0.001
ApoA1/	Carrier	1.72±0.02	1.67±0.03	1.76±0.05	1.66±0.07		
ApoB	Noncarrier	1.69±0.05	1.75±0.03	1.70±0.02	1.71±0.02		
	*P*	0.499	0.005	0.199	0.475	0.129	0.155

TC, total cholesterol; TG, triglyceride; HDL-C, high-density lipoprotein cholesterol; LDL-C, low-density lipoprotein cholesterol; ApoA1, apolipoprotein A1; ApoB, apolipoprotein B; ApoA1/ApoB, the ratio of apolipoprotein A1 to apolipoprotein B. The value was presented as median (interquartile range). The difference among the genotypes was determined by the Kruskal-Wallis test or the Wilcoxon-Mann-Whitney test.

### Correlation between genotypes and serum lipid parameters

Multiple linear regression analysis showed that the –1131T>C genotypes were correlated with serum TG levels, and c.553G>T and c.457G>A genotypes were associated with serum HDL-C levels in nondrinkers (*P*<0.05 for all). For drinkers, the –1131T>C genotypes were correlated with serum TC, TG, LDL-C, ApoB levels and the ratio of ApoA1 to ApoB (*P*<0.01 for all); c.553G>T genotypes were correlated with serum TC, TG, HDL-C and LDL-C levels (*P*<0.05–0.01); and c.457G>A genotypes were associated with serum TG, LDL-C, ApoA1 and ApoB levels (*P*<0.05–0.01; Table 5). Serum lipid parameters were also correlated with age, sex, height, weight, BMI, alcohol consumption, cigarette smoking, and blood pressure in both nondrinkers and drinkers (Table 6).

**Table 5:** Correlation between serum lipid parameters and alleles/genotypes in the nondrinkers and drinkers.

Lipid	Correlative factor	Unstandardized coefficient	Standard error	Standardized coefficient	*t*	*P*
Nondrinker and drinker						
TC	ApoA5 c.457G>A genotype	–0.183	0.076	–0.070	–2.404	0.016
	ApoA5 c.553G>T genotype	–0.248	0.114	–0.065	–2.184	0.029
TG	ApoA5 –1131 T>C allele	0.297	0.073	0.119	4.067	0.000
	ApoA5 c.457G>A genotype	–0.853	0.383	–0.248	–2.231	0.026
HDL-C	ApoA5 c.553G>T genotype	–0.207	0.058	–0.107	–3.569	0.000
LDL-C	ApoA5 –1131 T>C genotype	0.079	0.032	0.074	2.476	0.013
	ApoA5 c.457G>A genotype	–0.137	0.057	–0.071	–2.383	0.017
ApoA1	ApoA5 c.457G>A genotype	–0.047	0.012	–0.112	–3.872	0.000
ApoB	ApoA5 –1131 T>C genotype	0.034	0.010	0.103	3.534	0.000
	ApoA5 c.457G>A genotype	–0.040	0.017	–0.068	–2.312	0.021
ApoA1/ApoB	ApoA5 –1131 T>C allele	–0.087	0.031	–0.084	–2.809	0.005
Nondrinker						
TG	ApoA5 –1131 T>C allele	0.177	0.081	0.095	2.198	0.028
HDL-C	ApoA5 c.553G>T genotype	–0.170	0.077	–0.094	–2.214	0.027
	ApoA5 c.457G>A genotype	–0.111	0.055	–0.084	–2.000	0.046
Drinker						
TC	ApoA5 –1131 T>C genotype	0.265	0.058	0.198	4.607	0.000
	ApoA5 c.553G>T genotype	–0.340	0.154	–0.095	–2.200	0.028
TG	ApoA5 –1131 T>C genotype	0.320	0.091	0.143	3.502	0.001
	ApoA5 c.457G>A genotype	–1.414	0.504	–0.363	–2.804	0.005
	ApoA5 c.553G>T genotype	0.500	0.244	0.084	2.052	0.041
HDL-C	ApoA5 c.553G>T genotype	–0.262	0.084	–0.132	–3.112	0.002
LDL-C	ApoA5 –1131 T>C genotype	0.140	0.046	0.134	3.067	0.002
	ApoA5 c.457G>A genotype	–0.202	0.078	–0.111	–2.598	0.010
	ApoA5 c.553G>T genotype	–0.282	0.121	–0.101	–2.335	0.020
ApoA1	ApoA5 c.457G>A genotype	–0.062	0.015	–0.178	–4.202	0.000
ApoB	ApoA5 –1131 T>C genotype	0.059	0.013	0.192	4.579	0.000
	ApoA5 c.457G>A genotype	–0.051	0.022	–0.096	–2.279	0.023
ApoA1/ApoB	ApoA5 –1131 T>C genotype	–0.126	0.028	–0.185	–4.490	0.000

TC, total cholesterol; TG, triglyceride; LDL-C, low-density lipoprotein cholesterol; ApoA1, apolipoprotein A1; ApoB, apolipoprotein B.

**Table 6:** Correlation between serum lipid parameters and several environmental factors in the nondrinkers and drinkers.

Lipid	Correlative factor	Unstandardized coefficient	Standard error	Standardized coefficient	*t*	*P*
Nondrinker and drinker						
TC	Body mass index	–0.409	0.073	–1.098	–5.614	0.000
	Age	0.014	0.002	0.253	8.586	0.000
	Weight	0.211	0.032	1.915	6.623	0.000
	Height	–0.122	0.020	–1.221	–6.171	0.000
	Sex	0.322	0.066	0.168	4.844	0.000
TG	Weight	0.410	0.041	2.849	9.958	0.000
	Height	–0.250	0.026	–1.910	–9.730	0.000
	Body mass index	–0.846	0.094	–1.738	–8.959	0.000
	Sex	0.262	0.088	0.105	2.976	0.003
	Alcohol consumption	0.160	0.058	0.088	2.751	0.006
	Diastolic blood pressure	0.008	0.004	0.062	2.055	0.040
HDL-C	Alcohol consumption	0.139	0.023	0.198	6.027	0.000
	Age	0.003	0.001	0.099	3.187	0.001
	Sex	0.106	0.031	0.111	3.380	0.001
	Weight	–0.004	0.002	–0.081	–2.455	0.014
	Diastolic blood pressure	0.003	0.002	0.067	2.101	0.036
LDL-C	Body mass index	0.052	0.008	0.192	6.460	0.000
	Age	0.007	0.001	0.185	6.308	0.000
ApoA1	Alcohol consumption	0.066	0.007	0.302	9.701	0.000
	Age	0.001	0.000	0.170	5.811	0.000
	Sex	0.046	0.009	0.153	4.980	0.000
ApoB	Body mass index	0.019	0.002	0.233	7.968	0.000
	Age	0.003	0.000	0.209	7.262	0.000
ApoA1/ApoB	Body mass index	–0.030	0.006	–0.150	–4.912	0.000
	Age	–0.003	0.001	–0.106	–3.513	0.000
Nondrinker						
TC	Age	0.016	0.002	0.320	7.825	0.000
	Body mass index	0.078	0.015	0.212	5.160	0.000
	Cigarette smoking	–0.212	0.068	–0.127	–3.096	0.002
TG	Diastolic blood pressure	0.015	0.004	0.156	3.576	0.000
	Cigarette smoking	–0.179	0.068	–0.115	–2.635	0.009
HDL-C	Sex	0.212	0.041	0.226	5.169	0.000
	Age	0.004	0.001	0.154	3.637	0.000
	Height	–0.004	0.002	–0.096	–2.172	0.030
LDL-C	Body mass index	0.074	0.011	0.287	7.083	0.000
	Age	0.010	0.001	0.276	6.920	0.000
ApoA1	Sex	0.054	0.016	0.166	3.424	0.001
	Age	0.002	0.000	0.243	5.932	0.000
	Body mass index	0.014	0.004	0.238	3.716	0.000
	Cigarette smoking	–0.033	0.012	–0.124	–2.607	0.009
ApoB	Body mass index	0.023	0.003	0.286	7.080	0.000
	Age	0.003	0.000	0.291	7.326	0.000
ApoA1/ApoB	Body mass index	–0.029	0.009	–0.140	–3.182	0.002
	Age	–0.004	0.001	–0.144	–3.323	0.001
Drinker						
TC	Weight	0.026	0.006	0.227	4.483	0.000
	Age	0.007	0.003	0.118	2.675	0.008
	Sex	0.189	0.087	0.104	2.180	0.030
TG	Body mass index	–1.340	0.257	–2.149	–5.217	0.000
	Weight	0.650	0.110	3.397	5.930	0.000
	Height	–0.424	0.074	–2.288	–5.706	0.000
HDL-C	Body mass index	–0.037	0.009	–0.180	–4.181	0.000
	Diastolic blood pressure	0.005	0.002	0.104	2.431	0.015
	Age	0.003	0.001	0.086	1.996	0.046
ApoA1	Age	0.001	0.000	0.136	2.986	0.003
	Height	0.002	0.001	0.103	2.315	0.021
	Body mass index	–0.007	0.002	–0.120	–2.773	0.006
	Diastolic blood pressure	0.002	0.001	0.111	2.553	0.011
ApoB	Body mass index	0.012	0.004	0.141	3.361	0.001
ApoA1/ApoB	Body mass index	0.264	0.081	1.387	3.252	0.001
	Height	0.090	0.023	1.592	3.835	0.000
	Weight	–0.126	0.035	–2.158	–3.635	0.000

TC, total cholesterol; TG, triglyceride; LDL-C, low-density lipoprotein cholesterol; ApoA1, apolipoprotein A1; ApoB, apolipoprotein B.

## Discussion

In the present study, we show that the levels of TC, TG, HDL-C, ApoA1 and ApoB were lower in nondrinkers than in drinkers. There was no significant difference in the levels of LDL-C and the ratio of ApoA1 to ApoB between the two groups. These results are consistent with those of our previous studies [2], [3], [5]. Light to moderate alcohol consumption has been shown to protect against the development of CAD and mortality [7], [8], the dose-response relation between alcohol consumption and risk of CAD is J- or U-shaped [4], suggesting that the risk of CAD is greatest when alcohol consumption is high. The protective effects of regular, light to moderate alcohol consumption on CAD have been attributed to high serum HDL-C and ApoA1 levels [9]–[11], [58], suppressed coagulation capacity of platelets, or the suspected role of anti-oxidant substances contained in alcoholic beverages [59]. According to Rimm et al. [58], a daily dose of 30 g alcohol results in an average HDL-C level rise of 3.99 mg/dl, and an ApoA1 level rise of 8.82 mg/dl. The harmful effects of heavy alcohol consumption on serum lipid profiles may be due to an increase in plasma TG levels [13], [58], [60]. Alcohol can cause an increase of TG lipase activity and a decrease of the HDL removal from the circulation [12]. In a previous meta-analysis, 30 g of alcohol daily was associated with a plasma TG increase of 5.69 mg/dl [58]. The alcohol intake of 60 g/day increases the TG levels by about 0.19 mg/dl per 1 gram of alcohol consumed [13].

The present study shows that there was no significant difference in the allelic and genotypic frequencies of the three SNPs in ApoA5 gene between the nondrinkers and drinkers. The frequency of the –1131C allele in our study populations was similar to that in Chinese (29.9%) [27], [48], Singaporean Chinese (29.4%) [22], Malays (30.0%) [22], slightly lower than that in Japanese (34.0%) [18], [21], but much greater than that of whites (8.0%) [16], Hispanic Americans (16.0%) [20], [23], [26] or Tunisian (13.0%) [34]. The frequency of the c.553T allele in our study is extremely low, and is in agreement with that of two previous studies in Chinese (3.97%) [50] and Chinese Taiwanese (4.2–7.2%) [31], [49]. The c.553T allele has been reported to be absent in Caucasians [61]. The c.553TT homozygous was not detected in our study subjects. This is similar to the results in a previous study [50]. The frequency of c.457A allele was lower in our subjects than in Chinese Taiwanese (10.27%) [48], but was higher than that reported for Czechoslovakians in whom the c.457A allele frequency was 2.04% [62]. These results suggest that there exists significant racial/ethnic variation of allelic frequencies in ApoA5 gene.

The association of ApoA5 gene polymorphisms and plasma or serum lipid levels in humans has been evaluated in a large number of studies [20]–[44]. However, previous findings are inconsistent [52], [53]. Several separate clinical studies have provided consistent and strong support for the effect with 24% of whites, 35% of blacks, and 53% of Hispanics who carry –1131C allele associated with increased plasma TG levels [16], [20], [23], [63]. But this association was not significant in a population-based Spanish control group [53]. The ApoA5 c.553G>T polymorphism has been found to correlate strongly with TG levels in Chinese but not in Caucasians [31], [61]. The c.553T allele carriers had significantly higher plasma TG levels as compared to the wild-type GG genotype, in both CAD and control groups. In a previous study, however, Tang et al. [50] did not find any significant associations between the c.553G>T polymorphism and plasma lipid parameters including TC, LDL-C, HDL-C, ApoA1, and ApoB in Han Chinese recruited from Jiangsu Province, People's Republic of China. The association of ApoA5 c.457G>A polymorphism and plasma lipid levels in humans has not been fully elucidated. In a previous study, Kao et al. [31] showed that the ApoA5 c.457G>A polymorphism was not associated with serum TG levels in normal people. In another recent study, Hubacek et al. [62] showed that the impact of statin treatment on lipid parameters did not significantly differ between carriers of the genotypes defined by the ApoA5 c.457G>A polymorphisms. In the present study, we showed a significant association between ApoA5 gene polymorphisms and some serum lipid parameters. Haplotype analysis with all three SNPs further supports the strong association between ApoA5 gene polymorphisms and serum lipid levels in our study subjects.

The interactions of ApoA5 gene polymorphisms and alcohol consumption on serum lipid levels are not fully known. In the present study, we detected an interaction between –1131T>C genotypes and alcohol consumption on serum ApoB levels and the ratio of ApoA1 to ApoB, between c.553G>T genotypes and alcohol consumption on serum LDL-C levels and the ratio of ApoA1 to ApoB, and between c.457G>A genotypes and alcohol consumption on serum TG levels. These findings suggest that some serum lipid parameters in our study subjects were partly influenced by the interactions of ApoA5 gene polymorphisms and alcohol consumption. Correlational studies have suggested that the relation between alcohol consumption and cardiovascular disease may be influenced by the type of alcohol drunk [64]. A number of prospective cohort studies have suggested that wine drinkers are at lower risk of death from all causes of cardiovascular disease than users of other alcoholic beverages [65]–[67]. However, the effect of different types of alcoholic beverages on serum lipid profiles is not well known. Several epidemiological studies found that regular consumption of small to moderate amounts of alcoholic beverages, regardless of the type, resulted in significantly greater levels of HDL-C [68]–[70], HDL_3_-C, and ApoA1 [70] in both males and females. In two previous studies, however, Ruidavets et al. [71] found that wine was positively associated with HDL-C. Beer was positively associated with HDL-C in men and with TG in men and women. When taking drinking patterns into account, wine drinkers had higher HDL-C levels than non-wine drinkers. While Parker et al. [72] showed that both beer and liquor were independently associated with increased HDL-C in the total group, in men, and in women after covariates were controlled for. Wine was associated with a significant increase in HDL-C in women only. Choudhury et al. [9] also showed serum TG levels were significantly lower in those who drank beer. In our current study, 90% of the wine drunk by the subjects was corn wine, rice wine or rum, in which the alcohol content is low. Thus, the interactions of ApoA5 gene polymorphisms and different kinds of alcohol consumption on serum lipid levels still need to be determined.

There are several potential limitations in the present study. First, the sample size in both groups is a bit small. The individual with ApoA5 c.553TT genotype is not detected in our population, and the number of subjects with ApoA5 c.457AA genotype in both groups is also small. It has been postulated that an adequate analysis of the polymorphic variants of the ApoA1/ApoC3/ApoA4 gene complex requires a sample of at least 600 subjects to allow the detection of a twofold increased risk of disease [73]. Second, the ratio of male to female, mean age, blood pressure levels, and the percentages of subjects who smoked cigarettes were higher in drinkers than in nondrinkers. Although sex, age, BMI, blood pressure, and cigarette smoking have been adjusted for the statistical analysis, we can not completely exclude the influence of these factors on serum lipid levels among different genotypes in both groups. Third, the interactions of ApoA5 gene polymorphisms and cigarette smoking on serum lipid levels were not investigated in this study. In several previous epidemiological studies, we have shown that there was no significant correlation between cigarette smoking and serum lipid parameters or the prevalence of hyperlipidemia in our population [2], [3], [74]. Fourth, the diet and physical activities were not adjusted for the statistical analysis. In the present study, the population (Hei Yi Zhuang) is a special and isolated ethnic subgroup of the Zhuang minority in China. The population size is 51,655. Because of isolation from the other ethnic groups, the special customs and cultures including their clothing, intra-ethnic marriages, diet and lifestyle are still completely conserved to the present day. The diet in this population is consistent throughout the year and among individuals because of the Hei Yi Zhuang's reliance on a limited number of locally available food items. Great majority of Hei Yi Zhuang people live in the mountainous areas. The staple food is corn gruel or corn tortillas. On ordinary days, they are vegetarians [2], [3], [70]. In addition, all of the subjects were rural agricultural workers. The overall physical activity in the subjects was similar. Finally, it is well known that serum lipid levels are affected by multiple environmental and genetic factors, and their interactions. Although we have detected the interactions of three ApoA5 SNPs and alcohol consumption on serum lipid levels in this study, there are still many unmeasured environmental and genetic factors and their interactions. Thus, the interactions of gene-gene, gene-environment, and environment-environment on serum lipid levels remain to be determined.

### Conclusion

The present study shows that there was no significant difference in the genotypic and allelic frequencies of ApoA5 –1131T>C, c.553G>T and c.457G>A polymorphisms between the nondrinkers and drinkers. But the interactions of ApoA5 gene polymorphisms and alcohol consumption on serum lipid levels are different among the genotypes. The interactions between –1131T>C genotypes and alcohol consumption on serum ApoB levels and the ratio of ApoA1 to ApoB, between c.553G>T genotypes and alcohol consumption on serum LDL-C levels and the ratio of ApoA1 to ApoB, and between c.457G>A genotypes and alcohol consumption on serum TG levels were detected in the present study. The differences in some serum lipid parameters between the drinkers and nondrinkers might partly result from different interactions of the ApoA5 gene polymorphisms and alcohol consumption.
